# New Insights into Histidine Triad Proteins: Solution Structure of a *Streptococcus pneumoniae* PhtD Domain and Zinc Transfer to AdcAII

**DOI:** 10.1371/journal.pone.0081168

**Published:** 2013-11-28

**Authors:** Beate Bersch, Catherine Bougault, Laure Roux, Adrien Favier, Thierry Vernet, Claire Durmort

**Affiliations:** 1 Institut de Biologie Structurale, Université Grenoble Alpes, Grenoble, France; 2 Institut de Biologie Structurale, Direction des Sciences du Vivant, Commissariat à l′Energie Atomique et aux Energies Alternatives, Grenoble, France; 3 Institut de Biologie Structurale, Centre National de la Recherche Scientifique, Grenoble, France; University of Saskatchewan, Canada

## Abstract

Zinc (Zn^2+^) homeostasis is critical for pathogen host colonization and invasion. Polyhistidine triad (Pht) proteins, located at the surface of various streptococci, have been proposed to be involved in Zn^2+^ homeostasis. The *phtD* gene, coding for a Zn^2+^-binding protein, is organized in an operon with *adcAII* coding for the extracellular part of a Zn^2+^ transporter. In the present work, we investigate the relationship between PhtD and AdcAII using biochemical and structural biology approaches. Immuno-precipitation experiments on purified membranes of *Streptococcus pneumoniae (S. pneumoniae)* demonstrate that native PhtD and AdcAII interact *in viv*o confirming our previous *in vitro* observations. NMR was used to demonstrate Zn^2+^ transfer from the Zn^2+^-bound form of a 137 amino acid N-terminal domain of PhtD (t-PhtD) to AdcAII. The high resolution NMR structure of t-PhtD shows that Zn^2+^ is bound in a tetrahedral site by histidines 83, 86, and 88 as well as by glutamate 63. Comparison of the NMR parameters measured for *apo*- and Zn^2+^-t-PhtD shows that the loss of Zn^2+^ leads to a diminished helical propensity at the C-terminus and increases the local dynamics and overall molecular volume. Structural comparison with the crystal structure of a 55-long fragment of PhtA suggests that Pht proteins are built from short repetitive units formed by three *β*-strands containing the conserved HxxHxH motif. Taken together, these results support a role for *S. pneumoniae* PhtD as a Zn^2+^ scavenger for later release to the surface transporter AdcAII, leading to Zn^2+^ uptake.

## Introduction

Transition metals are essential for cell viability as they specifically bind to structural or catalytic metal sites in many proteins. On the other hand, excess free metals are toxic as they can catalyze the production of free radicals or may displace other metals from their cognate binding sites. Therefore, intracellular metal concentrations are tightly regulated through the controlled expression of metallochaperones, metal importers, storage proteins, and metal efflux or detoxifying proteins (for a recent review see Reyes-Caballero *et al.*, 2011[1]). Zn^2+^ can act both as a structural and catalytic cofactor [2], [3]. It has been shown that Zn^2+^ plays an important role in host pathogen interactions. While its uptake is important for bacterial virulence [4], [5], [6], this metal is also essential for the efficiency of the adaptive and cognate immune response of the host [4], [7].

In bacteria, Zn^2+^ homeostasis is achieved by the interplay of Zn^2+^ uptake and efflux. Zn^2+^ uptake is generally achieved via ABC (ATP Binding Cassette) transporters or solute carrier proteins (ZIP, ZrT- and IrT-like proteins, SLC39A) [8].

In *Escherichia coli (E. coli)*, Zn^2+^ uptake involves the high-affinity Tro-like ABC transporter ZnuABC and a low-affinity transporter ZupT, belonging to the ZIP family [9], [10], [11]. In the Znu system, ZnuA is the periplasmic Zn^2+^-binding protein. Zn^2+^-specific TroA-like proteins, such as ZnuA, contain a flexible, histidine-rich loop that has been suggested to play a role in the management of Zn^2+^ by a so far unknown mechanism [12], [13], [14]. Under conditions of Zn^2+^ depletion, expression of *znuABC* is up-regulated by the Zn^2+^-sensor Zur, a member of the Fur family [15]. In addition to the ABC transporter, the Zur-regulated ZinT protein (formerly YodA) has been described as an auxiliary component of ZnuABC, required at extreme Zn^2+^ deficiency [10], [12], [16], [17], [18]. *Salmonella* Typhimurium (*S.* Typhimurium) ZinT has been found to form a stable complex with ZnuA *in vitro* and to restore efficient Zn^2+^ import in *Salmonella* mutants expressing a modified ZnuA protein lacking the histidine-rich flexible loop [12].

Zn^2+^ homeostasis in the Gram-positive bacterium *Bacillus subtilis (B. subtilis)* has been described in detail by the Helmann group [19]. Here, Zn^2+^ uptake is achieved by the Zur-regulated ABC transporter AdcABC (formerly YcdH, YceA, YcdI), an ortholog of ZnuABC in Gram-negative bacteria. In addition, the ZinT-like YrpE protein has also been shown to belong to the Zur regulon [20]. In *B. subtilis*, Zn^2+^ uptake is further connected to oxidative stress via the PerR-regulated ZosA Zn^2+^ -uptake protein, a P-type ATPase [21].

Members of the Streptococcaceae (the genera Streptococcus and Lactococcus) generally regulate Zn^2+^ uptake by MarR-like transcriptional repressors instead of Zur, found in most other bacteria. These proteins are known as AdcR or ZitR in Streptococcus or Lactococcus, respectively [20], [22], [23]. Microarray analysis was performed in order to determine the AdcR regulon in *S. pneumoniae* [22]. The latter includes genes encoding for the Zn^2+^-specific ABC uptake system AdcABC, for a second extracellular Zn^2+^-binding protein, AdcAII, and for the streptococcal polyhistidine triad proteins (PhtA, B, D, and E) [22], [24], [25], [26]. The extracellular Zn^2+^-binding proteins AdcA and AdcAII have been shown to be redundant in Zn^2+^ uptake [27]. While AdcAII closely resembles *E. coli* ZnuA (23% sequence identity), AdcA corresponds to a fusion of a ZnuA- and a ZinT-like protein [28]. Interestingly, the *adcAII* gene is in an operon with the gene encoding the polyhistidine triad protein PhtD [24]. The two corresponding proteins were also found to co-localize at the surface of *S. pneumoniae* and to interact *in vitro* [29]. These observations strongly suggest that PhtD and AdcAII are functionally related.

Polyhistidine triad (Pht) proteins are streptococcal surface proteins that contain multiple copies of a characteristic HxxHxH sequence, designed as histidine triads, which were predicted to bind divalent metal cations [20], [30]. Proteins belonging to this family have recently been identified in different streptococci [31], [32]. *S. pneumoniae* is however the only microorganism to express as much as four Pht proteins, whose sequences are very similar especially in their N-terminal domains. Even though their individual physiological functions are not yet fully understood, their highly immunogenic character defines them as potential vaccine antigens [33], [34]. Indeed, administration of PhtA, PhtE or PhtD (also referred to as HtpS in *Streptococcus suis*) protects mice against *S. pneumoniae* colonization of the nasopharynx and against bacteremia by inducing a humoral response [35], [36], [37]. In this context, PhtD appears especially attractive because it shows the uppermost efficacy of protection in nasopharyngeal colonization models and displays the highest level of conservation among *S. pyogenes*, *S. agalactiae*, *S. suis* and *S. pneumoniae* [37], [38], [39] as well as across pneumococcal serotypes [40], [41]. In addition, single *phtD* deletion in *S. pneumoniae* induces significant attenuation in intranasal challenge model [25] suggesting its involvement in the pathogenesis of pneumococcal diseases.

The PhtD protein contains five histidine triad motifs (HxxHxH), predicted to bind divalent metal cations. The following experimental results directly demonstrated Zn^2+^-binding to Pht proteins: (i) HtpA (a PhtD homologue from *S. pyogenes*) was purified using a Zn^2+^-bound NitriloTriacetic Acid (NTA) column [42]; (ii) Zn^2+^ was detected by Induced Coupled Plasma Mass Spectrometry (ICP-MS) analyses of full length and truncated *S. pneumoniae* PhtD [29], suggesting that each of the five histidine triads in the full-length protein chelated a single Zn^2+^ ion; (iii) the affinity for Zn^2+^ of the 137 amino acid N-terminal domain of PhtD (t-PhtD, amino acids 30 to 166) was determined by isothermal titration calorimetry (*K_d_ ≈* 100 nM) [29]; (iv) the crystal structure of a short 55-residue fragment of PhtA (amino acids 166 to 220) revealed a HxxHxH motif in complex with Zn^2+^ [43].

Despite the increasing amount of biochemical, structural and physiological data, the functional role of PhtD and the other Pht proteins remains unclear. PhtD could be involved in AdcAII-mediated Zn^2+^-uptake as supported by the observation that the growth of a quadruple Δ*phtABDE S. pneumoniae* mutant was impaired unless supplemented with Zn^2+^ and Mn^2+^ [40]. Alternatively, Pht proteins could play a role in protecting the pneumococcus from toxic effects of high Zn^2+^ concentrations by scavenging, storing or trapping Zn^2+^ ions. Zn^2+^-binding to PhtD could also play a conformational role in relation to a so far unknown function as recently proposed by Plumptre [32]. Therefore, a more detailed structural and biochemical characterization of PhtD is required to unravel the functional relationship between PhtD and AdcAII proteins.

In the present work, we investigate this relationship using biochemical and structural biology approaches. We show that native PhtD and AdcAII proteins interact at the surface of *S. pneumoniae*. The 137 amino acid-long N-terminal domain of PhtD (t-PhtD, amino acids 30 to 166), that contains a single histidine triad motif, is studied by NMR spectroscopy. We demonstrate that Zn^2+^ is bound by the histidine triad of t-PhtD and that, in presence of *apo*-AdcAII, the metal ion is transferred from Zn^2+^-t-PhtD to AdcAII. We solve the high-resolution NMR structure of the Zn^2+^-bound form of t-PhtD. This structure provides new key structural data for this protein family for which only a structure of a short, 55-residue fragment was known. Detailed comparison of the two available structures of Pht proteins reveals a short conserved motif characteristic of streptococcal HxxHxH-containing proteins.

## Materials and Methods

### Plasmids

Plasmids were constructed as previously described [24], [29]. The pLIM02-*adcAII* and pLIM09-t-*phtD* plasmids were used for the expression of the AdcAII (residues Gly28 to Lys311) and the t-PhtD (residues Gly30 to Ser166) proteins, respectively. Recombinant proteins were fused to a His_6_-tag at the N-terminus. A tobacco etch virus protease (TEV) cleavable site (ENLYFQG) was inserted between the His_6_-tag and the N-terminal sequence of the proteins.

### Protein production and purification

Recombinant unlabeled t-PhtD and AdcAII proteins were produced in *E. coli* strain BL21(DE3)RIL in LB medium as described previously [24], [29].

*U*-^15^N-labeled AdcAII and t-PhtD proteins were produced by culturing the transformed *E. coli* strains BL21(DE3)RIL in M9 minimal medium pH 7.2, supplemented with glucose (4 g/L), 0.1 mM MnCl_2_, 0.05 mM FeCl_3_, 0.05 mM ZnSO_4_, a vitamin solution, 30 mg/L kanamycin or 100 µg/mL ampicillin, and ^15^NH_4_Cl (1 g/L) (Cambridge Isotope Laboratories) [44]. For *U*-^15^N,^13^C-labeled t-PhtD protein, ^13^C_6_-glucose (2 g/L) (Euriso-Top) was used as carbon source. Protein expression was induced with 0.5 mM isopropyl-β-D-1-thiogalactopyranoside (IPTG) overnight at 27°C. After cell disruption, the soluble recombinant proteins were loaded onto a NiNTA-agarose column. Lysis and column equilibration were performed using 50 mM HEPES (pH 8), 150 mM NaCl, 20 mM imidazole. After extensive washing, recombinant proteins were eluted with 20 to 300 mM imidazole gradient. After cleavage of the His_6_-tag with the TEV protease, both AdcAII and t-PhtD proteins were concentrated and dialyzed against a 50 mM HEPES (pH 8), 50 mM NaCl buffer (buffer A). For NMR resonance assignment and structure determination, *apo*- or Zn^2+^-t-PhtD samples were prepared in 50 mM MES buffer, pH 6.3.

### Preparation of the *apo*-form of the recombinant proteins

To generate the *apo*-forms of AdcAII and t-PhtD, the proteins were extensively dialyzed against buffer A containing 50 mM EDTA followed by extensive dialysis against buffer A containing 3.4 g/L chelex 100 resin (BioRad) to remove any trace of metal ion. All subsequent experiments involving the *apo*-proteins were carried out in buffers previously treated with the chelex 100 resin. Use of classical M9 minimal medium without addition of metal ions was found to be essential for obtaining the *apo*-AdcAII protein. The metal content of ^15^N-labeled t-PhtD or AdcAII was determined from ^1^H,^15^N-HSQC spectra whereas the ^1^H-NMR spectra were used for the unlabelled proteins. In the case of unlabeled AdcAII, thermal shift assay was also used to check the metal content of the purified proteins using an IQ5 96-well format real-time PCR instrument (Bio-Rad). Briefly, 12.5 µg of *apo*- or Zn^2+^-AdcAII proteins were mixed with 2 µL of 100× Sypro Orange (Molecular Probes). Samples were heat-denatured from 20 to 100°C at a rate of 1 °C per minute. Protein thermal unfolding curves were monitored by detection of changes in fluorescence of the Sypro Orange. The inflection point of the fluorescence *versus* temperature curve was identified by plotting the first derivative. The minima of the derived curve were referred to as the melting temperatures. The fluorescence of buffers and salt solutions was checked as controls. Melting temperature values of *apo*- and Zn^2+^-AdcAII have previously been determined to 52 °C and 79 °C, respectively [24].

### Immuno-precipitation (IP)

Pneumococcal membranes were prepared in 50 mM HEPES, 150 mM NaCl buffer at pH 8 (buffer B) as described previously [29]. Membranes were solubilized in 2% Triton X-100 for 10 min at 4° C with agitation and the mixture was ultracentrifuged at 200,000 g for 30 min. Membranes (Mb) contained in the supernatant were immuno-precipitated with polyclonal antibody against AdcAII under stirring for 1 h, after which 50 µL of protein A sepharose beads were added and the mixture was stirred for 40 min. Sepharose beads were pelletted and washed three times in buffer B containing 2% Triton X-100 before boiling for 5 min in 70 µL 4× Laemmli buffer. The supernatant (IP) was recovered by centrifugation. When specified proteins were cross-linked before the immuno-precipitation steps through incubation of membrane preparations (1 mL) with the short thiol-cleavable cross-linking agent 3,3′-dithiobis(sulfosuccinimidyl-propionate) (DTSSP) (0.8 mM) for 1 h (x-IP). The cross-linking reaction was quenched by adding 250 µL of 2 mM Tris (pH 7). Samples (Mb: 2 µL, the third wash (W): 5 µL, IP and x-IP: 15 µL) were loaded onto a sodium dodecylsulfate polyacrylamide 12.5% gel for electrophoresis (SDS-PAGE) and were immuno-blotted using specific primary polyclonal antibodies from rabbit or mouse. Secondary α clean Blot IP Detection Agent 1/400 (Pierce) was used to reveal primary rabbit antibodies, whereas the horseradish peroxydase-conjugated goat anti-mouse IgG (1/100) was used to detect PhtD mouse primary antibody. The immuno-reactive bands were revealed using chemiluminescence detection with the ECL system (GE Healthcare). Signals recorded on autoradiography were quantified using the Image J software and used to calculate the ratios of immuno-precipitated proteins.

### NMR spectroscopy: general features

Most NMR experiments were performed on Varian VNMRS 600 or 800 MHz spectrometers equipped with triple-resonance (^1^H, ^13^C, and ^15^N) cryogenic probes and shielded z-gradients. Experiments for structural characterization were recorded at 308 K, with a typical protein concentration of 0.8 mM, unless otherwise stated. Proton chemical shifts were referenced with respect to the H_2_O signal at 4.68 ppm relative to DSS. ^13^C and ^15^N chemical shifts were referenced indirectly using the relative ^1^H:X gyromagnetic ratios according to Markley *et al.* [45]. NMR spectra were processed, referenced using NMRPipe [46], and visualized using either NMRView [47] or CcpNmr Analysis [48].

### NMR resonance assignments

Sequential backbone resonance assignments of *apo*- and Zn^2+^-t-PhtD in 50 mM MES buffer, pH 6.3 were performed at 308 K using a series of 3D experiments (HNCO, HNCA, CBCACONH, HNCACB) from the Varian Biopack. Zn^2+^-t-PhtD side chain resonances were assigned from ^1^H,^13^C-CT-HSQC, 3D-(H)CC(CO)NH, and ^15^N- and ^13^C-edited 3D-NOESY-HSQC experiments. Aromatic side chain resonances were assigned using a ^1^H,^13^C-CT-HSQC experiment centered on aromatic carbons and a 2D ^1^H,^1^H-NOESY experiment acquired in D_2_O buffered with 50 mM potassium phosphate, pH 6.3. The latter experiment was used to specifically link the side-chain of aromatic residues, including histidines, to the backbone *via* the two Hβ protons. A ^1^H,^15^N-SOFAST-HMQC experiment [49], modified for an optimal ^2^ *J*_HN_ coupling transfer according to Pelton *et al.* [50], was exploited for the assignment of the histidine aromatic nitrogen frequencies and allowed the identification of the three nitrogen atoms that coordinate Zn^2+^. All NOE experiments were performed using standard pulse sequences from the Varian Biopack with mixing times of 100 ms, and were used to extract distance constraints for the structure calculation protocol. In addition, a 3D ^13^C-methyl-selective NOESY-HSQC experiment [51] was performed with the same mixing time.

### NMR study of Zn^2+^ transfer between AdcAII and t-PhtD

Unlabelled and U-^15^N-labelled AdcAII and t-PhtD samples in the *apo*- and Zn^2+^-forms were dialyzed in the same buffer A before the NMR analysis. To study the protein-protein interaction, 250 µL of the 0.15–0.2 mM *U*-^15^N-labelled protein were placed in a Shigemi tube. Small amounts of the second, unlabelled protein were added stepwise and ^1^H,^15^N-HSQC spectra were recorded after each addition at different protein:protein ratios ranging from 0 to 5 at 308 K. Eight different protein-protein combinations were studied using this protocol: ^15^N-*apo*-AdcAII/*apo*-t-PhtD, ^15^N-*apo*-AdcAII/*Zn^2+^*-t-PhtD,^ 15^N-Zn^2+^-AdcAII/*apo*-t-PhtD, ^15^N-Zn^2+^-AdcAII/Zn^2+^-t-PhtD, *apo*-AdcAII/^15^N-*apo*-t-PhtD, *apo*-AdcAII/^15^N-*Zn^2+^*-t-PhtD, Zn^2+^-AdcAII/^15^N-*apo*-t-PhtD, Zn^2+^-AdcAII/^15^N-Zn^2+^-t-PhtD. Chemical shifts of the ^15^N-labeled protein were monitored along the titration for each combination. The specific chemical shift signature of each of the *apo* and Zn^2+^-bound proteins was used to determine the relative populations.

### NMR ^15^N-relaxation and protein dynamics

*R*_1_, *R*_1*ρ*_, and heteronuclear {^1^H}-^15^N NOE relaxation experiments were conducted at 600 MHz using a non-cryogenic probe and standard pulse sequences [52], [53]. Temperature was set to 308 K. During the *R*_1_ relaxation delay, cross-correlated relaxation was suppressed by applying a 550 µs cosine-modulated 180° squared pulse every 5 ms with an excitation maximum at 2 kHz from the carrier. *R*_1*ρ*_ was measured using a *B*_1_ field of 1500 Hz. Recycle delays were set to 3 s for *R*_1_ and *R*_1*ρ*_. Relaxation delays of 0.01, 0.02, 0.05, 0.09, 0.15, 0.25, 0.4, 0.6, 0.9, 1.2, 1.5, 1.8 s and 0.01, 0.02, 0.03, 0.05, 0.07, 0.09, 0.11, 0.13, 0.17, 0.25, 0.4 s were used to explore the magnetization decay for longitudinal and transverse relaxation, respectively. For the heteronuclear {^1^H}-^15^N-NOE, the amide proton signals were saturated with a 1.7 kHz WALTZ16 decoupling scheme centered at the amide proton frequency. The saturation and recycle delays were set to 3 and 5 s, respectively. NMRView was used to quantify peak intensities and relaxation rates were extracted with the Curvefit program (http://cpmcnet.columbia.edu/dept/gsas/biochem/labs/palmer/software/curvefit.html). Experimental data were fitted to a two-parameter single exponential and errors were estimated from a Monte-Carlo simulation, taking into account twice the root-mean-square noise of the spectra. Overlapping resonances were excluded from the analysis. Transverse relaxation rates (*R*_2_) were calculated from *R*_1*ρ*_ using the relation *R*_2_  =  (*R*_1*ρ*_- *R*_1_cos^2^(*θ*))/(1- cos^2^(*θ*)), with *θ*  =  tan^−1^(2πΔν/γ_N_B_1_) where Δν is the ^15^N resonance offset. The rotational diffusion tensor of Zn^2+^-t-PhtD was determined from the measured *R*_1_ and *R*_1*ρ*_-derived *R*_2_ relaxation rates of residues with a heteronuclear {^1^H}-^15^N-NOE ≥0.7, using the program Tensor2 [54]. For *apo*-t-PhtD, only residues 92–146 were considered in the Tensor2 analysis because their chemical shift did not vary as a function of the Zn^2+^-site occupancy.

### Structure calculation of Zn^2+^-t-PhtD

The UNIO'10 software [55] was used for automatic peak picking, initial NOE assignment, and distance restraint extraction. TALOS+-derived dihedral restraints [56] together with manually assigned long-distance restraints calculated from a 3D-methyl-selective ^13^C-NOESY experiment were used as additional input data. Initial structures were calculated using the Cyana molecular dynamics algorithm [57], [58] in the UNIO'10 software. In the initial calculations the Zn^2+^ ion and the definition of the Zn^2+^-binding site were omitted. Final structures were calculated using the ARIA 2.3.1/CNS 1.1 software [59], allowing for re-assignment of the peaks in the lists initially provided by UNIO'10. A Zn^2+^ ion was introduced by constructing a non-standard residue involving coordination to three histidines (residues H83, H86, H88) and one glutamic acid (E63), as described in the Supporting Information (see Protocol S1). A tetrahedral geometry of the Zn^2+^-site was assumed with bond-lengths of 2.0 Å for N-Zn^2+^ and O-Zn^2+^ in accordance with published crystal structures (Protein Data Bank, PDB codes 3KS3 and 2CS7). 1000 structures were calculated in the 8^th^ ARIA iteration from which 20 structures with the lowest total energy were selected and submitted to further refinement in explicit water to give the final structural ensemble.

## Results

### AdcAII and PhtD interact *in vivo*

**Figure 1:**
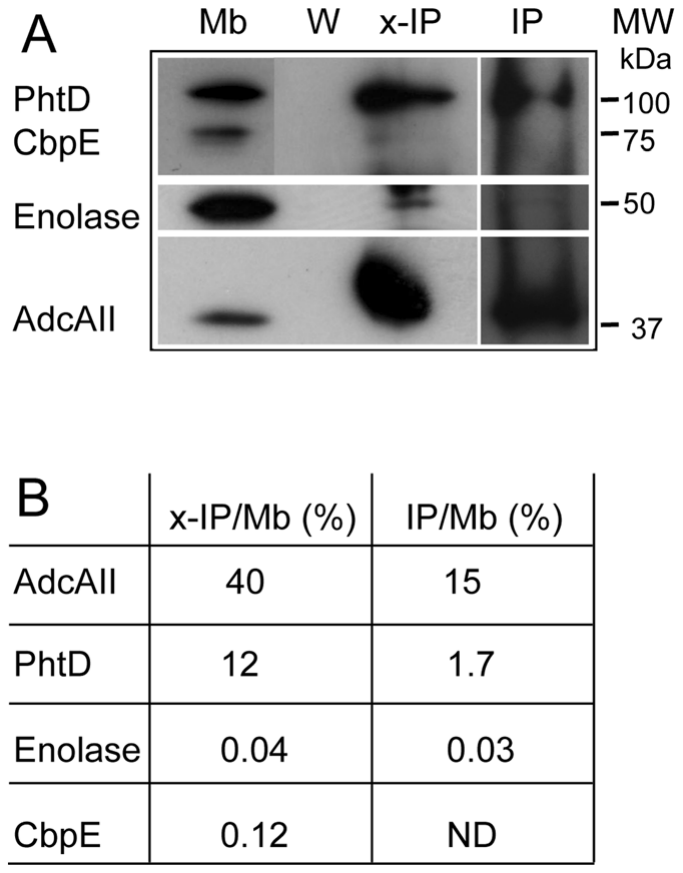
AdcAII and PhtD interaction *in vivo*. (A) Immuno-precipitation of AdcAII. *S. pneumoniae* D39 membranes (Mb) were immuno-precipitated with polyclonal antibody against AdcAII and protein A sepharose beads, then boiled in 4× Laemmli buffer. Membranes (Mb), the third wash (W), the immuno-precipitated fraction (IP), and the immuno-precipitated fraction from cross-linked membranes (x-IP) were loaded onto 12.5% SDS-PAGE, immuno-blotted by successive incubations with specific primary antibodies and revealed using chemiluminescent reagent. Exposure times of the films varied from 2 to 30 min depending on the antibody used. (B) Quantification of the ratio of immuno-precipitated proteins. Signals recorded on autoradiography were quantified using the Image J software. Absolute density values from the autoradiographies from the membrane (Mb) and the immuno-precipitated (IP and x-IP) fractions were used to calculate the percentage of enrichment in the immuno-precipitated fractions from a given membrane volume.

In a previous study, recombinant AdcAII was shown to interact *in vitro* with PhtD and with its derivative t-PhtD, i.e. the 137 amino acid-long N-terminal domain of PhtD including the first histidine triad (HxxHxH motif) [29]. AdcAII and PhtD were also observed to partially co-localize at the bacterial surface [29]. Therefore it was suggested that both proteins may also interact *in vivo*. To further support this hypothesis we performed immuno-precipitation of AdcAII from purified membranes of *S. pneumoniae*. One fraction of the membrane preparation was exposed to the thiol-cleavable cross-linking agent DTSSP prior to the immuno-precipitation, while another fraction was not. Western blot analysis (Fig. 1A) reveals that in both cases PhtD is co-precipitated with AdcAII. Other proteins located on the cell surface (Enolase and the choline-binding protein CbpE) were used as controls and were barely detectable in these analyses. The percentage of membrane-bound PhtD immuno-precipitated by AdcAII polyclonal antibodies increased from 1.7% to 12% for non-cross-linked (IP) versus cross-linked (x-IP) membranes, respectively (Fig. 1B). This suggests that the cross-linking agent stabilized the interaction. Analysis of the relative proportion of the various species shows that only 0.04% and 0.12% of the initial amounts of Enolase and CbpE were recovered in the x-IP fraction (Fig. 1B), whereas the corresponding level of PhtD was two orders of magnitude higher (12%), supporting an *in vivo* interaction between AdcAII and PhtD.

### Zn^2+^-transfer from t-PhtD to AdcAII

The previous immunoprecipitation results suggest that AdcAII and PhtD may be functionally related. Recently, Rioux et al. [40] have proposed that Pht proteins may act as metal ion scavengers for later release to surface transporters such as AdcAII when metals are in low abundance in the environment. In many metal trafficking systems studied, metal transfer involves formation of a metal-dependent protein complex [60], [61], [62].

**Figure 2:**
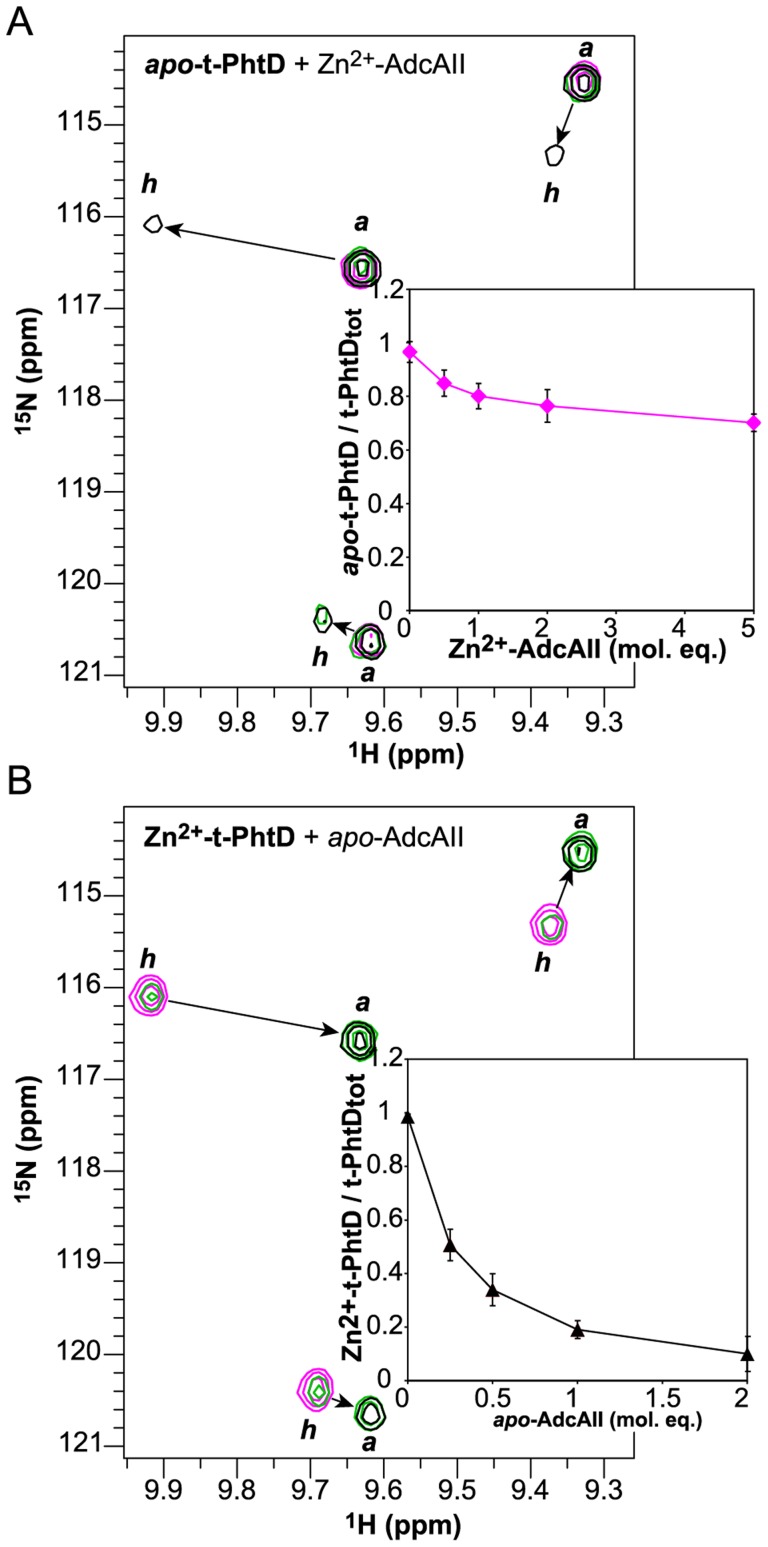
Zn^2+^ transfer between t-PhtD and AdcAII followed by NMR. 2D-^1^H,^15^N-HSQC experiments were used to monitor Zn^2+^ transfer between AdcAII and t-PhtD. Peak positions of the *apo* and *holo* forms are indicated by '*a*' and '*h*', respectively. (A) Titration of 0.13 mM *U*-^15^N-labeled *apo*-t-PhtD with unlabeled Zn^2+^-AdcAII. ^1^H,^15^N-HSQC spectra of *apo*-t-PhtD after addition of 0 (magenta), 2 (green), and 5 (black) molar equivalents of Zn^2+^-AdcAII. (B) Titration of 0.19 mM *U*-^15^N-labeled Zn^2+^-t-PhtD with unlabeled *apo*-AdcAII. ^1^H,^15^N-HSQC spectra of Zn^2+^-t-PhtD after addition of 0 (magenta), 0.5 (green), and 2 (black) molar equivalents of *apo*-AdcAII. This behavior is typical of a slow exchange process at the NMR timescale. The percentage of the two forms can thus be calculated from the peak volumes. Inserts show the evolution of the *apo*-t-PhtD (top) and Zn^2+^-t-PhtD populations (bottom) along the titration. The reported value is averaged over six individual pairs of resolved cross-peaks.

To check whether (A) Zn^2+^ transfer between AdcAII and PhtD can occur and (B) PhtD and AdcAII form a metal-dependent complex, NMR titration experiments were performed. In these experiments, t-PhtD was used as a model for full-length PhtD. ^1^H,^15^N-HSQC spectra of both AdcAII and t-PhtD show significant differences between the Zn^2+^-bound and the *apo* forms, allowing for quantification of the relative populations. Eight different ^1^H,^15^N-HSQC titration experiments were performed starting with *U*-^15^N-labeled t-PhtD or AdcAII both in the *apo*- or Zn^2+^-forms and adding the other, unlabelled *apo*- or Zn^2+^-protein partner. The spectrum of the labeled protein remained unaffected when the two *apo*- or Zn^2+^-loaded proteins were mixed (Supporting Fig. S1A-D). When *U*-^15^N-labeled *apo*-t-PhtD was titrated with unlabelled Zn^2+^-AdcAII, correlations characteristic of Zn^2+^-t-PhtD appeared at AdcAII/t-PhtD ratios of two to five indicating that a small fraction (roughly 20%) of t-PhtD protein acquired a Zn^2+^ ion (Fig. 2A). On the other hand, titration of *U*-^15^N-labeled Zn^2+^-t-PhtD with unlabeled substoechiometric quantities of *apo*-AdcAII induced formation of *apo*-t-PhtD (Fig. 2B). Within the limits of errors in the determination of protein and Zn^2+^ concentrations, the results of these experiments concordantly show that at AdcAII:t-PhtD:Zn^2+^ ratios of 1∶1∶1 between 80 and 100% of the Zn^2+^ ions is bound to AdcAII at equilibrium, whereas a five-fold molar excess of Zn^2+^-AdcAII over *apo*-t-PhtD is needed to convert roughly 20% of *apo*-t-PhtD to Zn^2+^-t-PhtD. Therefore it can be concluded that AdcAII has a higher Zn^2+^-affinity than t-PhtD and that Zn^2+^ is transferred from one protein to the other. No unidentified peaks appeared in any of these titration experiments, indicating that no stable protein complex was detected at equilibrium in the NMR spectra between either of the different forms of the two recombinant proteins (see Supporting Fig. S1).

Zn^2+^-transfer between the two proteins could occur *via* one of the following mechanisms: (A) Zn^2+^ dissociates from t-PhtD and the free Zn^2+^ ion is captured by AdcAII, this mechanism is non-specific and does not require a physical contact between the two proteins. (B) Zn^2+^-t-PhtD specifically interacts with *apo*-AdcAII and the Zn^2+^ ion is transferred within the complex, possibly *via* an intermediate with a metal coordination sphere shared between the two proteins. Such a mechanism has been demonstrated in the case of Cu^+^ transfer involving Cu^+^-ATPases [63]. The lifetime of the protein complex is determined by the relative kinetics of complex formation and dissociation. The formation of a thermodynamically stable complex between AdcAII and the N-terminal fragment of PhtD in significant proportion can be excluded from the present NMR titration study. However, the present NMR observations alone cannot distinguish between a direct, non-specific Zn^2+^-transfer (case A) and the formation of a transient complex allowing specific Zn^2+^-transfer between the two proteins (case B). To further investigate the mechanism of Zn^2+^-transfer between the two proteins, kinetic measurements were considered exploring the intrinsic fluorescence of the two tryptophane residues present in AdcAII. Nevertheless, the emission spectra of the *apo* and Zn^2+^-bound forms of AdcAII did not show any differences, thus precluding this type of study.

### High resolution NMR structure of Zn^2+^-t-PhtD reveals the metal binding site

To further characterize the biological function of the two proteins, a more detailed structural characterization of t-PhtD was undertaken. Indeed, to date, the only available high-resolution structure of a Pht protein is the X-ray crystal structure of a Zn^2+^-bound 54-residue fragment of *S. pneumoniae* PhtA, which includes the second histidine triad motif [43]. To gain additional high-resolution structural information on Pht proteins, which is lacking for this family of important virulence factors as outlined by Plumptre *et al.* [32], we relied on NMR. The structure of the truncated N-terminal domain of PhtD (t-PhtD, residues 30-166 of the unprocessed protein sequence), which is a stable fragment obtained by limited proteolysis [29], was determined in its Zn^2+^-bound state. The sequence of this construct, which includes the first histidine triad of the full length PhtD, is highly conserved among different Pht proteins from *S. pneumoniae* (see sequence alignment, Fig. S2). Nearly complete backbone (98.7%) and side chain (non-hydrogens: 73.8%, hydrogens: 92.95%) resonance assignment was obtained for residues 40 to 158 using standard 3D experiments as described in the Materials and Methods Section. Unassigned residues from the N- and C-termini (residues 30–39 and 159–166, respectively) were discarded from the structure calculation, and the final structural ensemble comprises only residues S40 to Q158 of the Zn^2+^-t-PhtD protein.

The Zn^2+^-binding site was built using information from protein sequence comparison and experimental data. The crystal structure of PhtA from *S. pneumoniae* shows that Zn^2+^ is bound to the three histidines of the second histidine triad and to an additional aspartate, located 21 residues upstream of the histidine motif [43]. Sequence alignment of streptococcal histidine triad proteins suggested E63, H83, H86 and H88 of the first histidine triad motif of PhtD as the Zn^2+^ ligands. The three nitrogen atoms that coordinate Zn^2+^ were experimentally identified according to the chemical shifts of the corresponding histidine imidazole C*δ*2 [64], N*δ*1 or N*ε*2 [65]. Whereas H83 and H86 unambiguously bind Zn^2+^ through their N*ε*2, Nδ1 is the Zn^2+^-ligand in the case of H88. The Zn^2+^ site was introduced in the final structure calculation of Zn^2+^-t-PhtD by constructing a non-standard residue, as described in the Materials and Methods section.

**Figure 3:**
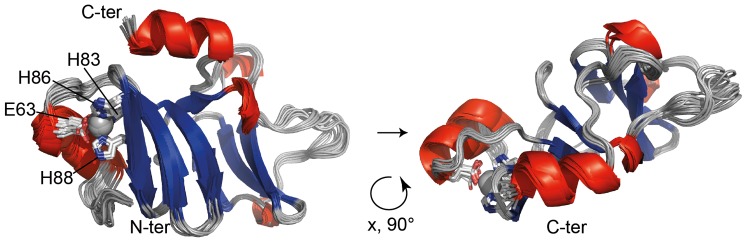
Zn^2+^-t-PhtD solution structure. Superposition of the 20 structures of the final ensemble in ribbon representation. *α*-helices are represented in red, *β*-sheets in blue. Side chains of Zn^2+^-coordinating residues (E63, H83, H86, H88) are shown as sticks and a pale grey sphere represents the Zn^2+^ ion. Residues 40 to 50 were found to be disordered and are not represented in this figure. The illustration was prepared using the PyMOL Molecular Graphics System, Version 1.5.0.4 Schrödinger, LLC.Pymol.

**Figure 4:**
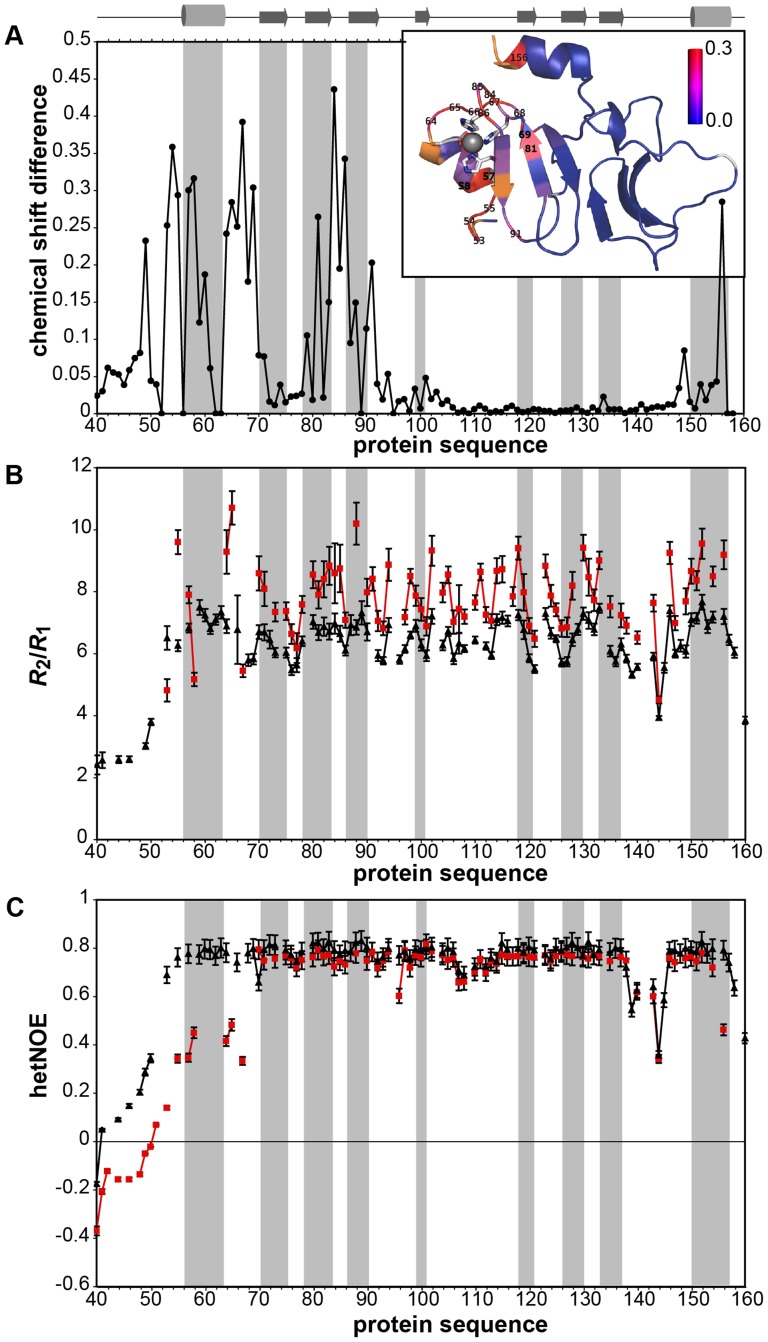
Comparison of *apo*- and Zn^2+^-t-PhtD. (A) Weighted chemical shift difference determined for the amide resonances between the two forms (Δδ  =  [(Δδ^1^H)^2^+(0.01 Δδ^15^N)^2^]^1/2^). The insert shows a color-coded projection of the chemical shift differences onto the ribbon structure of Zn^2+^-t-PhtD. Residues for which no resonance could be identified in the ^1^H,^15^N-HSQC spectrum of *apo*-t-PhtD are shown in orange and proline residues in white. Residues with significant chemical shift differences are identified by their residue numbers. (B) Ratio of transverse (*R*_2_) and longitudinal (*R*_1_) relaxation rates and (C) {^1^H}^15^N-heteronuclear NOE for *apo*- (red squares) and Zn^2+^-t-PhtD (black triangles). Secondary structure elements identified in the structure of Zn^2+^-t-PhtD are indicated on the top of the figure and by grey shadows in each of the panels.

The final structure calculation was performed with Aria/CNS using 2574 distance restraints and 186 TALOS+-derived dihedral angle restraints after inclusion of the Zn^2+^ coordination geometry. The final structural ensemble comprises 20 water-refined structures and is shown in Fig. 3 with an emphasis on the Zn^2+^ site. The backbone is very well defined with a root mean square deviation (rmsd) of 0.56 Å (residues 51–158) with respect to the mean structure. Zn^2+^-t-PhtD contains two nearly perpendicular *β*-sheets (sheet I: residues I70–T75, G78–H83, H86–Y90, I99–S101; sheet II: residues I118–E121, Y126–V130, K133–Y137) flanked by an N-terminal and a C-terminal helix (residues P56–E63 and K150–K157, respectively), the latter folding back to the top of *β*-sheet I. The two *β*-sheets are connected through a long loop that appears ordered throughout the structural ensemble. An additional short stretch of *α*-helical structure is formed by residues E102–L104. The Zn^2+^ coordinating residues are located at the end of the N-terminal helix (E63) and in the first *β*-sheet (H83, strand 2, H86 and H88 strand 3). Residues S40 to K50 do not adopt a well-defined structure within the ensemble, suggesting that this part of the protein is disordered. This was confirmed by the heteronuclear {^1^H}^15^N-heteronuclear NOE data (see below, Fig. 4C). Structural statistics are presented in Table 1. Structure calculations of Zn^2+^-t-PhtD in absence of Zn^2+^ converged to very similar structures (rmsd for the backbone atoms of the two ensemble averages: 0.79 Å), indicating that the presence of the non-standard residue did not influence the outcome of the structure calculation. Structure calculation of *apo*-t-PhtD was not attempted due to *apo*-t-PhtD inherent flexibility (see below).

**Table 1:** NMR structural statistics for Zn^2+^-t-PhtD.

**I. Experimental constraints**	
Distance restraints	
Unambiguous distance restraints	
total number	2574
intraresidual	1090
sequential	571
short range	231
medium range	124
long range	558
Ambiguous distance restraints	91
Dihedral angle restraints	
phi	93
psi	93
**II. Constraint violations**	
Distances	
rms	0.044 Å
largest violation	1.67 Å
Dihedral angles	
rms	1.16 °
largest violation	14.9 °
**III. Geometry**	
Mean deviation from ideal geometry	
bond lengths	0.005 Å
bond angels	0.660 °
impropers	1.766 °
**IV. Structure quality**	
Rmsd with respect to mean structure (residues 51–158)	
backbone	0.56 Å
heavy atoms	1.01 Å
CING ROG analysis (residues 51–158)^a^	
red	27%
orange	28%
green	45%
Ramachandran analysis (residues 51–158)	
core	86.1%
allowed	12.0%
generous	1.6%
disallowed	0.2%

a acording to iCING analysis [81]

### Zn^2+^ binding modifies protein structure and dynamics

Backbone chemical shifts are valuable probes not only for detecting changes in the chemical environment of a given nucleus but also for identifying secondary structure elements. Nearly complete backbone assignment was obtained for *apo*- and Zn^2+^-t-PhtD, allowing comparison of the two forms. Fig. 4A shows the weighted chemical shift difference between the two forms determined for backbone amide groups as a function of the protein sequence, while the insert sketches these differences plotted on the 3D-structure of Zn^2+^-t-PhtD. Chemical shift variations are essentially localized close to the Zn^2+^-binding site and at the end of the spatially close C-terminal helix. Several residues in these regions do not show cross-peaks in the ^1^H,^15^N-HSQC spectrum of the *apo*-form, presumably due to line broadening (namely E52, R62, E63, Y89, K157, Q158, indicated in orange in the insert of Fig. 4A). Analysis of the chemical shift data with TALOS+ reveals shortening of helix-1 in the *apo*-form of t-PhtD, whereas the *β*-strand structures appear conserved in the absence of Zn^2+^. Together, these observations not only suggest conformational differences between both forms at the Zn^2+^-binding site, but also the presence of additional backbone or side chain motions in the case of *apo*-t-PhtD.

In order to quantify differences in backbone motional behavior between *apo*- and Zn^2+^-t-PhtD, ^15^N-relaxation data were collected on the individual forms. *R*_1_ rates and heteronuclear NOEs are sensitive to high-frequency motions (nano- to picosecond timescales) whereas *R*_2_ rates (derived from the measured *R*_1*ρ*_rates) also sample much slower processes (micro- to millisecond timescales). Fig. 4B shows the ratio of transverse over longitudinal relaxation rate constants (*R*_2_/*R*_1_) for *apo*- and Zn^2+^-t-PhtD as a function of the protein sequence. Significantly increased *R*_2_/*R*_1_ ratios are observed in the *apo*-form for individual residues (T57, G64, I65 and H88), suggesting the presence of slow conformational exchange. In addition, comparison of {^1^H}^15^N-heteronuclear NOEs measured on the *apo*- and the Zn^2+^-protein (Fig. 4C) also shows significantly different values for residues in helix 1 and the preceding N-terminal stretch. {^1^H}^15^N-heteronuclear NOE sample backbone motion on a fast time scale and values lower than 0.5, as seen for residues of the *apo*-protein located in the region corresponding to helix 1, indicate conformational disorder. Therefore it can be concluded that the Zn^2+^ ion confers a higher stability to helix 1 with respect to the core of the protein domain.

For residues belonging to the protein core (i.e. those situated in secondary structure elements) the mean value of *R*_2_/*R*_1_ is proportional to the overall rotational tumbling of the protein and therefore to the molecular size. Comparing the data for the two different forms of t-PhtD, it can be seen that the *R*_2_/*R*_1_ ratio is systematically higher for *apo*-t-PhtD than for the Zn^2+^-form (Fig. 4B). The program Tensor2 [54] was used for a quantitative analysis of the relaxation rate constants measured for each of the two forms and the rotational diffusion tensor was determined using the molecular coordinates of the representative Zn^2+^-t-PhtD structure. Rotational diffusion of Zn^2+^-t-PhtD could best be fitted assuming completely anisotropic diffusion (*D*_xx_  = 1.99×10^7^ s^−1^, *D*_yy_ = 2.18×10^7^ s^−1^, *D*_zz_ = 2.59×10^7^ s^−1^). This corresponds to a harmonic mean rotational correlation time (*τ*_c_) of 7.4 ns. An identical value was calculated for *τ*_c_ from the molecular coordinates of Zn^2+^-t-PhtD using the program HydroNMR [66], suggesting that Zn^2+^-t-PhtD is a monomer in solution. According to Fig. 4A, residues 92–146 do not undergo significant chemical shift changes between the *apo* and Zn^2+^-bound form. These residues were therefore used in the Tensor2 program for the analysis of *apo*-t-PhtD dynamics. The principal values of the rotational diffusion tensor of the *apo* form are slightly smaller (*D*_xx_ = 1.72×10^7^ s^−1^, *D*_yy_ = 1.938×10^7^ s^−1^, *D*_zz_ = 2.39×10^7^ s^−1^) corresponding to a mean *τ*_c_ of 8.3 ns. This suggests either an equilibrium between monomeric and higher oligomeric species or an increased molecular volume of *apo*-t-PhtD that is consistent with the unfolding of the N-terminal *α*-helix. In any case, the increase in rotational correlation time of *apo*-t-PhtD is very modest, thus excluding significant protein oligomerization at the experimental concentration of 0.8 mM.

Based on our results, we suggest that loss of the Zn^2+^ ion leads to detachment of helix 1 from the core of the protein domain. PhtD helical propensity is thus diminished, while local dynamics and overall molecular volume are increased. The conformation of the core of the molecule, including the first *β*-sheet does not seem to be affected except for a possible increase in mobility of aromatic side chains, presumably Zn^2+^-binding histidines 83, 86 and 88, at the interface between helix 1 and the first *β*-sheet.

### PhtD:40–158 and PhtA:166–220 share common structural features

**Figure 5:**
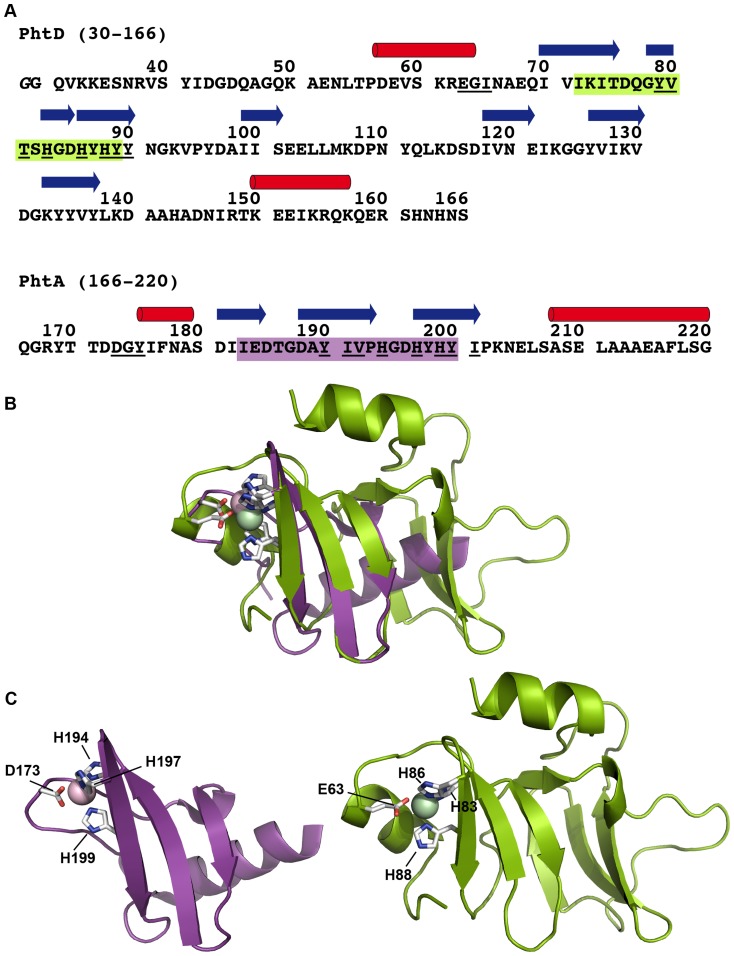
Comparison of the NMR structure of Zn^2+^-t-PhtD and the X-ray crystallographic structure of Zn^2+^-PhtA. (A) Protein sequences of the two Pht fragments for which three-dimensional structures are known. The PhtD fragment (this study) involves the first histidine triad motif of the Pht family whereas the PhtA fragment (2cs7.pdb, [43]) contains the second histidine triad motif. Red cylinders (*α*-helices) and blue arrows (*β*-strands) indicate experimentally determined secondary structure elements. Residues used for the structural alignment are shaded. Underlined letters identify residues that belong to the PROSITE motif proposed for the identification of histidine triad repeats (see text). (B) Superposition of Zn^2+^-t-PhtD (green) and Zn^2+^-PhtA (violet) structures. Shaded residues in the two Pht sequences are overlayed, as a basis for the superimposition. (C) Zn^2+^-t-PhtD and Zn^2+^-PhtA in the same orientation as in the superimposition. Amino acids bound to Zn^2+^ and the Zn^2+^ ion are shown as sticks and as a sphere, respectively.

The solution structure of Zn^2+^-t-PhtD is composed of two three-stranded *β*-sheets and two helices. The three histidines (H83, H86, and H88) of the histidine triad are located in the first sheet. Structural comparison with all protein structures deposited in the Protein Data Bank (PDB) using the DALI server [67] gave a single hit, the 55-residue fragment of the closely related pneumococcal PhtA protein (PDB code 2CS7 [43]). This fragment corresponds to PhtA residues 166–220, involves the second histidine triad, and is conserved in the PhtD protein (see sequence alignment, Supporting Fig. S2). The PhtA crystal structure also contains a single Zn^2+^ ion chelated by N*δ*1 of H194, N*ε*2 of H197, N*ε*2 of H199 and the carboxylate of D173. As in our Zn^2+^-t-PhtD structure, the three histidine ligands belong to a three-stranded *β*-sheet, formed by residues 181–184, 188–193, and 196–201. Surprisingly, the most significant structural alignment between the two protein structures, as suggested by the DALI algorithm, is obtained between the unique *β*-sheet in PhtA and the second *β*-sheet of t-PhtD which does not contain a histidine triad. Indeed, the C*α*, C′, N atoms of residues PhtA:172–201 and t-PhtD:109–138 can be superimposed with an rmsd of 2.27 Å (Supporting Fig. S3). However, in t-PhtD, the conserved histidine triad is located within the first and not the second *β*-sheet with a significant sequence similarity between the two histidine triad motifs. Therefore, a new structure superposition was created, considering this time the conserved secondary structure elements around the histidine triad of both proteins. The C*α*, C′, N atoms of residues PhtA:183–200 and t-PhtD:72–89 could be aligned with an rmsd of 1.07 Å, as shown in Fig. 5. While only the backbone atoms of residues located in the *β*-strands were used for the structural alignment, the relative orientation of the side chains forming the Zn^2+^-binding site is well conserved. In both metal sites the Zn^2+^ ion is chelated by two N*ε*2 and one N*δ*1 atoms of the three histidine side chains, and a carboxylate provided by an aspartate in the case of PhtA and a glutamate in the case of PhtD. According to the sequence alignment of the four different Pht proteins from *S. pneumoniae* D39 (Supporting Fig. S2), the acidic residue (Asp or Glu) is conserved.

### Full-length Pht proteins are presumably formed from repetitions of three stranded *β*-sheets connected by flexible linkers

Analysis of the distribution of secondary structure elements in PhtD, either from the solved structures or from predictions (Supporting Fig. S4), reveals that there are seven occurrences of three consecutive *β*-strands approximately 20 residues in length. Five of these repeats contain histidine triads (residues 70–90, 197–214, 302–322, 547–566, and 631–650), two do not (residues 118–137, and 388–409). This suggests that the full-length Pht proteins are built from these short repetitive units connected by loops of variable lengths. We further analyzed the PhtD sequence in order to obtain insight into the overall organization of these structured domains within the whole protein. The IUPred server [68] was used to screen the PhtD sequence for disorder propensity; the results are shown in Supporting Fig. S5. IUPred predicts five globular domains (residues 1–35, 56–143, 186–218, 444–570, 616–667). All of these, except for the first that corresponds to the signal peptide, contain histidine triad motifs. The region 302–322 also containing a histidine triad is not counted as a globular domain but the corresponding residues appear at the limit defined between order and disorder. Apart from these domains, there are long stretches of residues with a high, predicted disorder propensity. The presence of flexible regions within the Pht proteins is corroborated by the proteolysis of full-length recombinant proteins observed for PhtD [29] and PhtA [43].

### A new Prosite motif for the identification of streptococcal histidine triad containing proteins

It is interesting to note that the region of structural homology between the two protein fragments, involving each a different histidine triad motif (the first *vs.* the second one) is much more limited than originally proposed. Indeed, comparing the two structures of histidine triad containing protein fragments it becomes obvious that only the three-stranded *β*-sheet is conserved whereas the helical structures in both fragments are not. On the other hand, current identification of histidine triad-containing proteins uses the HMM (Hidden Markov Models) built from sequence alignments that form the basis either of the PFAM [69] family entry PF04270 or the TIGRFAM [70] entry TIGR01363. Both signatures are also referenced in the InterPro [71] domain prediction database under the identifiers IPR006270 and IPR023832, respectively. Whereas the underlying sequence alignment of PF04270 is based on the crystal structure of PhtA, and includes more than 10 residues of low sequence complexity that form the C-terminal helix in PhtA (not conserved in the t-PhtD structure), the one of TIGR01363 includes the N-terminal half (348 residues) of streptococcal histidine triad proteins. Based on the structural alignment presented in Fig. 5, we looked for an alternative motif for the identification of protein sequences containing the streptococcal histidine triad. Retained criteria concern sequence conservation in the shorter protein fragment including conserved residues from the Zn^2+^-binding site and the surrounding *β*-sheet and led to the development of a new PROSITE [72] motif: [DEH]-G-[YILVTFMW]-x(12,16)-[YILVTFMW]-[YILVTFMWA]-[YILVTFMW]-x-H-x(2)-H-x-H-[YILVTFMW]-[YILVTFMW] (for the PROSITE pattern syntax see http://prosite.expasy.org/scanprosite/scanprosite_doc.html). This motif was used to scan the UniProtKB/Swiss-Prot (release 2012_10 of 31-Oct-12), UniProtKB/TrEMBL (release 2012_10 of 31-Oct-12) databases after addition of a criterion excluding sequences with less than two hits. This search yielded identification of 3922 hits in 1035 sequences. The taxonomic distribution of the resulting sequences reveals 4 sequences from the Genus *Gemella* and 1031 sequences from *Lactobacillales*, involving 2 and 3 sequences from *Aerococcus* and *Carnobacteriaceae* families, respectively, and 1026 sequences from the different serotypes of streptococci. The number of recovered sequences in the latter search is higher than the 915 sequences associated with PFAM PF04270 family. As already stated before, the majority of these sequences are Pht-like proteins (group 1 and group 3 in the classification of Plumptre *et al.* [32]) and internalin A-like proteins, containing leucine-rich repeats at the C-terminal, such as the previously described Blr and Slr proteins [38], [39] (see Table 2). Two *S. suis* proteins annotated as DNA gyrase/topo II, topoisomerase IV A subunits (SSU05_1267 and SSU98_1281) were identified to be Pht-like proteins due to their sequence similarity with other Pht-like proteins. Moreover, scanning the complete UniProtKB/Swiss-Prot and UniProtKB/TrEMBL databases against the newly designed PROSITE motif exclusively yielded sequences from firmicutes, suggesting its robustness against false positives. Thus, the above structural determination together with structural alignment leads to identify the shorter conserved motif of the histidine triad containing proteins.

**Table 2:** Genes related to AdcA and AdcAII as well as genes identified using the histidine triad PROSITE motifa,b.

Strain	AdcA-like	AdcAII-like	Pht-like	Internalin A-like
*S. agalactiae* A909	SAK_0685 (adcA)	SAK_1319 SAK_1898	SAK_1318 SAK_1897	SAK_1023^d^
*S. agalactiae* NEM316	gbs0580	gbs1307 (lmb) gbs1926	gbs1306 gbs1925^c^ gbs1924^c^	gbs0918
*S. dysgalactiae* subsp. *equisimilis* (strain GGS_124)	SDEG_0674 (znuA)	SDEG_0935	SDEG_0936	
*S. equi* subsp. *zooepidemicus* (MGCS10565)	Sez_0736 (znuA)	Sez_1736 (lraI)	Sez_1735 Sez_1790	Sez_0679 (inlA)
*S. mutans* UA 159	SMU.1302	-	-	-
*S. pneumoniae* TIGR4	SP2169	SP1002	SP1003 (phtD) SP1004 (phtE) SP1174 SP1175	
*S. pneumoniae* D39	SPD_1997	SPD_0888 (lmb)	SPD_0889 (phtD) SPD_0890 (phtE) SPD_1037 SPD_1038 (phpA) SPD_0892^c^	
*S. pyogenes* SF370	Spy_0714 (adcA)	Spy_2007 (lmb)	Spy_2006	Spy_1361 (inlA)
*S. pyogenes* Manfredo	SpyM51261	SpyM51680	SpyM51679	SpyM50750
*S. pyogenes* MGAS10394	M6_Spy0563	M6_Spy1717	M6_Spy1716	M6_Spy1083
*S. sanguinis* SK36	SSA_0138	SSA_1340 SSA_1990	SSA_1339 (phpA) SSA_1991 (phtA)	
*S. suis* 05ZYH33	SSU05_0112^g^	SSU05_0330^f^	SSU05_0332^f^ SSU05_1267^e^	SSU05_1577
*S. suis* 98HAH33	SSU98_0115	SSU98_0326	SSU98_0327 SSU98_1281^e^	SSU98_1587^c^
*S. thermophilus* LMD-9	STER_0895	–	–	–

a PROSITE motif: [DEH]-G-[YILVTFMW]-x(12,16)-[YILVTFMW]-[YILVTFMWA]-[YILVTFMW]-x-H-x(2)-H-x-H-[YILVTFMW]-[YILVTFMW].

b Genes are identified by their ordered locus names.

c C-terminal truncation.

d N-terminal truncation.

e TrEMBL Annotation/Description: Type IIA topoisomerase (DNA gyrase/topo II, topoisomerase IV), A subunit.

f SSU05_0330 and SSU05_0332 are adjacent ORFs in the same reading frame (+1). SSU05_0331 is a 93 bp fragment in a different reading frame (-1), annotated as bifunctional acetylornithine aminotransferase and succinyldiaminopimelate aminotransferase (fragment).

g this gene has been described to be under the control of the Zur-like transcriptional repressor SSU05_0310 [82].

## Discussion

In this work, we show that the two pneumococcal surface proteins PhtD and AdcAII interact *in vivo* and we demonstrate that recombinant t-PhtD, containing a single histidine triad, can transfer a Zn^2+^ ion to *apo*-AdcAII. We then present the high-resolution structure of a 137 amino acid N-terminal domain of PhtD (t-PhtD) in which Zn^2+^ is bound to the first histidine triad motif of PhtD. We provide new structural data on this challenging protein family that are exploited in comparison with the previous structure of a PhtA fragment. We propose a new Prosite pattern for the identification of histidine triad motifs in protein sequences from the existing databases.

NMR titration experiments demonstrated that Zn^2+^ is preferentially transferred from t-PhtD to AdcAII but no stable complex between the two proteins in any state could be detected. On the other hand, direct contact between the full-length PhtD protein and AdcAII was evidenced by immunoprecipitation experiments on native membranes in the presence and absence of a cross-linking agent (DTSSP). This agent is expected to stabilize transient protein-protein complexes. Taken together, these data suggest that the PhtD protein could capture Zn^2+^ ions from the environment before shuttling them to the cell membrane-anchored lipoprotein AdcAII *via* formation of a transient, short-lived (at the NMR timescale) complex. Such a transfer could be required for Zn^2+^-uptake by AdcAII.

**Figure 6:**
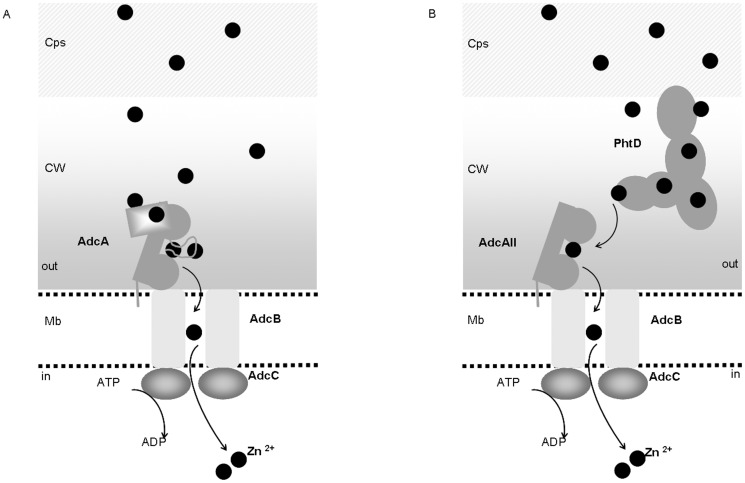
Model of Zn^2+^ transport in Streptococcus pneumoniae. (A) Representation of Zn^2+^ binding to the different domains of AdcA: the histidine rich loop, the ZinT-like C-terminal domain and the N-terminal AdcAII-like Zn^2+^-binding site. (B) Model of Zn^2+^-binding and Zn^2+^-transfer from PhtD to AdcAII in which the ZinT-like sequence and the histidine-rich loop are missing. Cps; capsular polysaccharides, CW: cell wall, Mb: membrane.

AdcA and AdcAII are redundant for Zn^2+^ uptake [27]. AdcA consists of a ZnuA- and a ZinT-like domain [28], the first of which possesses a characteristic histidine-rich loop. This protein thus contains multiple Zn^2+^-binding sites. The high affinity site in the N-terminal ZnuA-like domain is expected to be designed by three histidines and one glutamate, by analogy with known structures of ZnuA [13], [73], [74], [75] and AdcAII (or Lmb) [24], [76], [77]. AdcA may also accommodate an additional metal ion in the histidine-rich loop close to the N-terminal metal-binding site [12], [13], [14]. According to different ZinT crystal structures (4AYH, 1TXL, unpublished), the ZinT-like C-terminal domain of AdcA contains a high affinity Zn^2+^-site including H452, H461, H463. Additional Zn^2+^ sites have been described in ZinT [28] but their physiological relevance may be questioned [10]. Studies in *S.* Typhimurium show that either ZinT or the histidine-rich loop in ZnuA were required for efficient Zn^2+^-uptake [12]. Based on these analogies, we propose for AdcA-mediated Zn^2+^-uptake the model that is schematized in Fig. 6A. AdcAII lacks both, the ZinT-like domain and the histidine-rich loop [24] and may require different auxiliary proteins for efficient Zn^2+^-uptake. In *S. pneumoniae*, the role of PhtD could thus be analogous to that suggested for the ZinT-like supplementary domain of AdcA. An illustration of this hypothetical model is shown in Fig. 6B.

The underlying hypothesis of a functional relationship between both proteins could further be supported by the analysis of the genetic context in different streptococci. In fact, genes coding for PhtD-like proteins contiguous with those coding for a Zn^2+^-binding lipoprotein homologous to metal-binding receptors (AdcAII in *S. pneumoniae*) have been described in *S. pyogenes* [78], *S. pneumoniae* [29], and *S. suis* [79]. Therefore, the proteomes of different representative streptococcal species were scanned for Pht-, AdcA-, and AdcAII-like proteins. Pht-like proteins were identified using the PROSITE motif developed in the present work whereas AdcA- and AdcAII-like proteins were identified by a Blast search against the protein sequence of AdcAII from *S. pneumoniae* D39 (Q04KS9_STRP2, Lmb). AdcA- and AdcAII-like proteins were further differentiated according to their size. Whereas AdcAII-like proteins typically consist of approximately 300 residues, AdcA-like proteins have the additional ZinT-like domain discussed above, resulting in a protein size larger than 500 residues. The results of this scan are shown in Table 2. It can be seen that in each species that contains *pht*-like genes at least one of those is contiguous with an *adcAII*-like gene and *vice versa*. In some species, contiguous *pht*- and *adcAII*-like genes have even been identified in two copies. Remarkably, species lacking Pht-like proteins, such as *S. mutans* or *S. thermophilus*, also lack AdcAII-like proteins. This suggests that *pht*- and *adcAII*-like genes have been maintained as an operon during evolution and that the corresponding proteins are therefore functionally related. Phylogenetic analysis of Pht expression through evolution in various streptococci suggests a preponderant role of Pht proteins in streptococcus survival and pathogenic infection processes [80]. This could explain gene duplication that has been frequently observed. The functional redundancy of the four Pht proteins was pointed out by *in vivo* experiments [25]. No virulence attenuation occurred in D39 pneumococcus mutants lacking either a single or two Pht proteins with the exception of the *phtA-B* double mutant. On the other hand a mutant lacking the four Pht proteins was found to be completely avirulent. According to the authors of this study this is due to its inability to bind complement factor H. Recruitment of complement factor H limits complement deposition on the pneumococcal surface, thus leading to immune escape. Additional evidence for such a redundancy was corroborated in *S. pneumoniae* TIGR4 [40]. Indeed the growth curve of a *phtABDE* quadruple mutant was impaired unless supplemented with Zn^2+^ or Mn^2+^ whereas single or double Pht mutants showed no growth phenotypes in chemically defined medium supplemented or not with different bivalent metals.

The data presented in the present study, together with previous work, indicate that histidine triad motifs in PhtD do bind Zn^2+^ ions that could subsequently be transferred to AdcAII. Such a transfer could play a role in AdcAII-mediated Zn^2+^-import into the pneumococcus and suggests that *S. pneumoniae* PhtD is a Zn^2+^ scavenger for later release to the surface transporter AdcAII when the metal is in low concentration in the host environment, a mechanism involved in pneumococcus infectivity.

### Data Deposition

Chemical shift assignments, restraint lists and molecular coordinates have been deposited in the BioMagResBank (http://www.bmrb.wisc.edu/) under the accession number 18943 and the Protein Data Bank (http://www.ebi.ac.uk/pdbe/) under the accession number 3ZFJ.

## Supporting Information

Figure S1 **NMR titration experiments.** (A) ^15^N-labeled *apo* t-PhtD (0.06 mM) in presence of 5 mol. eq. *apo*-AdcAII (green). (B) ^15^N-apo-AdcAII (0.05 mM) in presence of 2 mol. eq. *apo*-t-PhtD (green). (C) ^15^N-Zn^2+^-t-PhtD (0.06 mM) in presence of 5 mol. eq. Zn^2+^-AdcAII (green). (D) ^15^N-Zn^2+^-AdcAII (0.1 mM) in presence of 1 mol. eq. Zn^2+^-t-PhtD (blue). (E) ^15^N-*apo*-AdcAII (0.1 mM) in presence of 1 mol. eq. Zn^2+^-t-PhtD (blue). (F) ^15^N-Zn^2+^-AdcAII (0.1 mM) in presence of 1 mol. eq. *apo*-t-PhtD (green). In figures S1A-S1F, the reference spectra of ^15^N-labelled apo and Zn^2+^-bound proteins are shown in red and black, respectively.
(TIF)

Figure S2 **Sequence alignment of PhtD, PhtE, PhtA, and PhtB (from top to bottom) of** ***S. pneumoniae*** **D39.** Conserved residues are shaded in blue, partially conserved residues in grey. PhtD residues 1-240 are numbered including the t-PhtD construct (residues 29–166) studied by NMR In the present work.
(EPS)

Figure S3 **Structural alignment of the NMR structure of Zn^2+^-t-PhtD and the crystal structure of Zn^2+^-PhtA proposed by the DALI server. Zn^2+^-t-PhtD is shown in green, Zn^2^-PhtA in violet.** Experimentally determined secondary structures are indicated in the sequence representation. Residues involved in the structural alignment are shaded on the individual sequences. Rmsd for C*α*, C′, N atoms of residues PhtA:172–201 and t-PhtD:108–138 = 2.27 Å.
(EPS)

Figure S4 **Experimentally determined and predicted secondary structure of PhtD.** Secondary structure elements determined by NMR and X-ray crystallography (PhtA structure, PDB code 2cs7.pdb) are shown in bold font. Predictions were obtained through the PSIPRED (Pred, Buchan D.W. *et al.*, Nucleic Acids Res. 2010;38(Web Server issue):W563-8) and the Jpred (Jpred, Cole C. *et al.*, Nucleic Acids Res. 2008;36(Web Server issue):W197–201) servers. H and E denote *α*-helices and *β*-strands, respectively. Possible Zn^2+^ sites were identified using the proposed PROSITE motif (see text) and are shaded in green. Note that it was not possible to identify the fourth ligand in the fifth HxxHxH motif due to sequence divergence.
(DOC)

Figure S5 **Prediction of globular and disordered domains from the PhtD sequence.** The program IUPred (Dosztányi, Z. *et al.*, Bioinformatics, 2005;21:3433–3434) was used to predict globular domains in PhtD. Top: globular domains are indicated in blue capital letters whereas residues in predicted disordered regions are shown in red lower-case letters. Bottom: disorder tendency as a function of the protein sequence as calculated by IUPred. Blue bars indicate predicted globular domains. The position of the fragments of known 3D structure is indicated in gray on the protein sequence.
(EPS)

Protocol S1 **This protocol describes the construction and the force-field parameters of the non-standard residue for the Zn^2+^-site using CNS.**
(DOC)
