# Supplementary figures and images for: New Insights into Histidine Triad Proteins: Solution Structure of a Streptococcus pneumoniae PhtD Domain and Zinc Transfer to AdcAII
Source: PLoS One. 2013 Nov 28;8(11):e81168. doi: 10.1371/journal.pone.0081168 (PMC3842936; doi:10.1371/journal.pone.0081168)

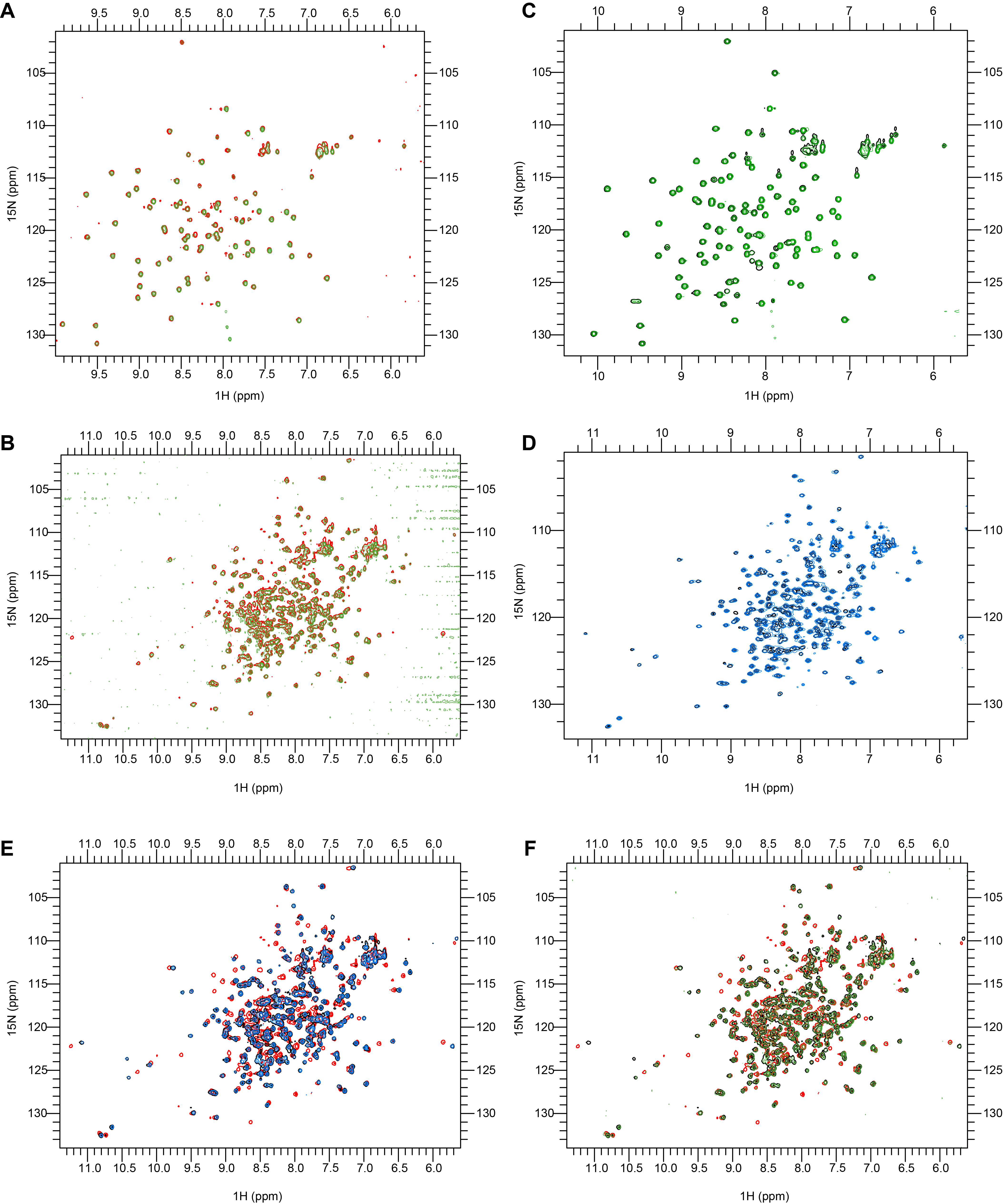

Supplement: Figure S1 — NMR titration experiments. (A) 15N-labeled apo t-PhtD (0.06 mM) in presence of 5 mol. eq. apo-AdcAII (green). (B) 15N-apo-AdcAII (0.05 mM) in presence of 2 mol. eq. apo-t-PhtD (green). (C) 15N-Zn2+-t-PhtD (0.06 mM) in presence of 5 mol. eq. Zn2+-AdcAII (green). (D) 15N-Zn2+-AdcAII (0.1 mM) in presence of 1 mol. eq. Zn2+-t-PhtD (blue). (E) 15N-apo-AdcAII (0.1 mM) in presence of 1 mol. eq. Zn2+-t-PhtD (blue). (F) 15N-Zn2+-AdcAII (0.1 mM) in presence of 1 mol. eq. apo-t-PhtD (green). In figures S1A-S1F, the reference spectra of 15N-labelled apo and Zn2+-bound proteins are shown in red and black, respectively. (TIF) [file pone.0081168.s001.tif]
