# Supplementary material for: New Insights into Histidine Triad Proteins: Solution Structure of a Streptococcus pneumoniae PhtD Domain and Zinc Transfer to AdcAII
Source: PLoS One. 2013 Nov 28;8(11):e81168. doi: 10.1371/journal.pone.0081168 (PMC3842936; doi:10.1371/journal.pone.0081168)
Supplement: Figure S4 — Experimentally determined and predicted secondary structure of PhtD. Secondary structure elements determined by NMR and X-ray crystallography (PhtA structure, PDB code 2cs7.pdb) are shown in bold font. Predictions were obtained through the PSIPRED (Pred, Buchan D.W. et al., Nucleic Acids Res. 2010;38(Web Server issue):W563-8) and the Jpred (Jpred, Cole C. et al., Nucleic Acids Res. 2008;36(Web Server issue):W197–201) servers. H and E denote α-helices and β-strands, respectively. Possible Zn2+ sites were identified using the proposed PROSITE motif (see text) and are shaded in green. Note that it was not possible to identify the fourth ligand in the fifth HxxHxH motif due to sequence divergence. (DOC) [file pone.0081168.s004.doc]

**PhtD (*S. pneumoniae* D39)**

Pred: ----HHHHHHHHHHHHHHHHHHHH-------------EEEEE----HHHH----------

Jpred ----EEHHHHHHHHHHHHHHHHHHH------------EEEEEE-----------------

**exp: PhtD (NMR): ----------------HHHHH**

AA: MKINKKYLAGSVAVLALSV-SYELGRHQAGQVKKESNRVSYIDGDQAGQKAENLTPDEVS

10 20 30 40 50 60

Pred: -HH------EEEEE---EEEEE----EEEE-------HHHHHHH--------------EE

Jpred ---------EEEEE---EEEE------EEE------------------------------

**exp: HHH------EEEEEE--EEEEEE--EEEEE--------EEE----------------EEE**

AA: KREGINAEQIVIKITDQGYVTSHGDHYHYYNGKVPYDAIISEELLMKDPNYQLKDSDIVN

70 80 90 100 110 120

Pred: E----EEEEE--EEEEEE----------HHHHHHH-------------HHHHHHHH----

Jpred -----EEEE----EEEEE------------HHHHH------------HHHHHHH------

**exp: E----EEEEE--EEEEE------------HHHHHHHH- PhtA (x-ray): ---**

AA: EIKGGYVIKVDGKYYVYLKDAAHADNIRTKEEIKRQKQERSHNHNSRADNAVAAARAQGR

130 140 150 160 170 180

Pred: --------------------EEEEE--------------HHHHHHHHHHH----------

Jpred ------EEE-----------EEEE----------------HHHHHHHH------------

**exp: ------------EEEE---EEEEEE--EEEEEE-----HHHHHHHHHHHHHH**

AA: YTTDDGYIFNASDIIEDTGDAYIVPHGDHYHYIPKSDLSASELAAAQAYWNGKQGSRPSS

190 200 210 220 230 240

Pred: --------------------------------HHHHHHHHH-------------------

Jpred -------------------------------HHHHHHHHHHH------------------

AA: SSSHNANPAQPRLSENHNLTVTPTYHQNQGENISSLLRELYAKPLSERHVESDGLIFDPA

250 260 270 280 290 300

Pred: ---------EEE------EEEE-----HHHHHHHH-------------------------

Jpred -EEE----EEEEE----EEEEE-------HHHHHHHH-----------------------

AA: QITSRTANGVAVPHGDHYHFIPYSQLSPLEEKLARIIPLRYRSNHWVPDSRPEQPSPQST

310 320 330 340 350 360

Pred: ----------------------HHHHHHHHEE----EEE---------------HHHHH-

Jpred ----------------------------EEEE----EEE-----EEEEE--------H--

AA: PEPSPSPQPAPNPQPAPSNPIDEKLVKEAVRKVGDGYVFEENGVPRYIPAKDLSAETAAG

370 380 390 400 410 420

Pred: ------------------------------------HHHHH-------------HHHHHH

Jpred HHHH-------------------------------HEE-----EEEE------HHHHHHH

AA: IDSKLAKQESLSHKLGAKKTDLPSSDREFYNKAYDLLARIHQDLLDNKGRQVDFEALDNL

430 440 450 460 470 480

Pred: HHHH-------HHHHHHHH----------------------HHHHHHHHH----------

Jpred HH---------EEE----HHHH---------------------HHHHHH-----------

AA: LERLKDVSSDKVKLVDDILAFLAPIRHPERLGKPNAQITYTDDEIQVAKLAGKYTTEDGY

490 500 510 520 530 540

Pred: --------------EE----------------HHHHHHHHHHHH----------------

Jpred ------EEE-----EEEE-----EEE----------HHHHHHH-----------------

AA: IFDPRDITSDEGDAYVTPHMTHSHWIKKDSLSEAERAAAQAYAKEKGLTPPSTDHQDSGN

550 560 570 580 590 600

Pred: -----HHHHHHH-------------------EEE---EEEE-------------------

Jpred ------HHH---------------------EEEE----EEEE-----EEE------HHHH

AA: TEAKGAEAIYNRVKAAKKVPLDRMPYNLQYTVEVKNGSLIIPHYDHYHNIKFEWFDEGLY

610 620 630 640 650 660

Pred: --------HHHHHHH----------------------HHH--------------------

Jpred -------HHHHHHHHHHHHH---------------HHHHHHH------------------

AA: EAPKGYSLEDLLATVKYYVEHPNERPHSDNGFGNASDHVQRNKNGQADTNQTEKPNEEKP

670 680 690 700 710 720

Pred: ------------------------------------------------HH----------

Jpred ------------------------------------------------------------

AA: QTEKPEEDKEHDEVSEPTHPESDEKENHVGLNPSADNLYKPSTDTEETEEEAEDTTDEAE

730 740 750 760 770 780

Pred: --------HHHHHHHHHHHHH----HHHHHHHHHHHHH-------------HHHHHHHHH

Jpred ------HHHHHHHHHHHHHHHHH---H-H--HHHHHHHH---EEEEEE-----HHHHHHH

AA: IPQVEHSVINAKIAEAEALLEKVTDSSIRQNAVETLTGLKSSLLLGTKDNNTISAEVDSL

790 800 810 820 830 840

Pred: HHHHHH-------

Jpred HHHHH--------

AA: LALLKESQPTPIQ

850
