# Supplementary material for: New Insights into Histidine Triad Proteins: Solution Structure of a Streptococcus pneumoniae PhtD Domain and Zinc Transfer to AdcAII
Source: PLoS One. 2013 Nov 28;8(11):e81168. doi: 10.1371/journal.pone.0081168 (PMC3842936; doi:10.1371/journal.pone.0081168)

**Supporting protocol S1.**

This protocol describes the construction and the force-field parameters of the non-standard residue for the Zn^2+^-site using CNS

**CNS input file generatemetal.inp:**

remarks file generate/generatemetal.inp

remarks Sample: generate protein structure with metal cluster

topology

@/data/people/bersch/aria/zn_phtd/generate_zn/topallhdg_Zn_5.3.pro

{*Append the metal cluster*}

autogenerate angles=true end {*topology. *}

mass ZN_1 65.390 {*from cns/libraries/toppar/ion.top*}

RESIdue ZN {zinc2+} {*from cns/libraries/toppar/ion.top*}

GROUp

ATOM ZN TYPE=ZN_1 CHARge=+2.0 END

END {ZN}

{*Generate the patch topology,*}

{*which will be used in the *}

{*PATCh command. *}

PRESidue HHHE ! 3 His +1 GLU , order HISD,HISD,HISE,GLU,ZN

DELETE ACCEPTOR 4OE2

DELETE DONOR 1HE2 1NE2

DELETE DONOR 2HE2 2NE2

DELETE DONOR 3HD1 3ND1

GROUP

DELETE ATOM 1HE2 END

MODIFY ATOM 1NE2 TYPE=NR CHARge=-0.990 END !Zn-coordination

MODIFY atom 1CG type=C5 charge= 0.130 end ! as in HISD patch

MODIFY atom 1ND1 type=NH1 charge=-0.570 end ! as in HISD patch

MODIFY atom 1HD1 type=H charge= 0.420 end ! as in HISD patch

MODIFY atom 1CD2 type=CR1E charge= 0.1 end ! as in HISD patch

MODIFY atom 1CE1 type=CRH charge= 0.410 end ! as in HISD patch

group

DELETE ATOM 2HE2 END

MODIFY ATOM 2NE2 TYPE=NR CHARge=-0.990 END !Zn-coordination

MODIFY atom 2CG type=C5 charge= 0.130 end ! as in HISD patch

MODIFY atom 2ND1 type=NH1 charge=-0.570 end ! as in HISD patch

MODIFY atom 2HD1 type=H charge= 0.420 end ! as in HISD patch

MODIFY atom 2CD2 type=CR1E charge= 0.1 end ! as in HISD patch

MODIFY atom 2CE1 type=CRH charge= 0.410 end ! as in HISD patch

group

DELETE ATOM 3HD1 END

MODIFY ATOM 3ND1 TYPE=NR CHARge=-0.990 END !Zn-coordination

MODIFY ATOM 3CG TYPE=C5 CHARge= 0.130 END ! as in HISE patch

MODIFY ATOM 3CE1 TYPE=CRH CHARge= 0.410 END ! as in HISE patch

MODIFY ATOM 3CD2 TYPE=CR1E CHARge= 0.100 END ! as in HISE patch

MODIFY ATOM 3NE2 TYPE=NH1 CHARge=-0.570 END ! as in HISE patch

MODIFY ATOM 3HE2 TYPE=H CHARge= 0.420 END ! as in HISE patch

group

MODIFY ATOM 4OE1 TYPE=O CHARge=-0.45 END ! as in GLN, +0.05 for total charge of -0.5

MODIFY ATOM 4OE2 TYPE=OZ CHARge=-0.8 END ! new TYPE, OZ. Charge as in GLU

MODIFY ATOM 4CD TYPE=C CHARge=0.75 END ! as in GLU, +0.05 for total charge of -0.5

MODIFY ATOM 4CG TYPE=CH2E CHARge=0.00 END ! as in GLU

add bond 5ZN 1NE2

add bond 5ZN 2NE2

add bond 5ZN 3ND1

add bond 5ZN 4OE2

add angle 1NE2 5ZN 2NE2

add angle 1NE2 5ZN 3ND1

add angle 1NE2 5ZN 4OE2

add angle 2NE2 5ZN 3ND1

add angle 2NE2 5ZN 4OE2

add angle 3ND1 5ZN 4OE2

add angle 5ZN 1NE2 1CE1

add angle 5ZN 1NE2 1CD2

add angle 5ZN 2NE2 2CE1

add angle 5ZN 2NE2 2CD2

add angle 5ZN 3ND1 3CE1

add angle 5ZN 3ND1 3CG

add angle 5ZN 4OE2 4CD

add improper 1NE2 5ZN 1CD2 1CE1

add improper 2NE2 5ZN 2CD2 2CE1

add improper 3ND1 3CG 3CE1 5ZN

END

end

parameter

@/data/people/bersch/aria/zn_phtd/generate_zn/parallhdg_Zn_5.3.pro

{*Append parameters for metal site*}

! eps sigma eps(1:4) sigma(1:4)

! (kcal/mol) (A)

! ---------------------------------------

NONBonded ZN_1 0.01 1.568 0.01 1.568 {*from cns/libraries/toppar/ion.param*}

nbonds {*This statement specifies the*}

atom cdie shift eps=1.0 e14fac=0.4 {*nonbonded interaction energy*}

cutnb=7.5 ctonnb=6.0 ctofnb=6.5 {*options. Note the reduced *}

nbxmod=5 vswitch {*nonbonding cutoff to save *}

end {*CPU time. *}

end

{*Split the coordinate file into*}

{*two files, one containing the *}

{*protein coordinates, the other*}

{*the metal cluster *}

{*coordinates. *}

{*First, generate protein.*}

segment

name="PhtD"

chain

@/programs/i386-linux/aria/2.3.1/cns/toppar/topallhdg5.3.pep

coordinates @zn_phtd_template.pdb

end

end

coordinates @zn_phtd_template.pdb

{*Now generate metal cluster.*}

segment

name="ZN"

chain

coordinates @zn_exp.pdb

end

end

coordinates @zn_exp.pdb

{*Now generate the *}

{*covalent links between the protein and the metal*}

patch HHHE

reference=1=( resid 83 )

reference=2=( resid 86 )

reference=3=( resid 88 )

reference=4=( resid 63 )

reference=5=( resid 159 )

end

write coordinates output=zn_phtd.pdb end

write structure output=zn_phtd.psf end

stop

**Associated forcefield parameters:**

BOND NR ZN_1 1000.000 {sd= 0.001} 2.000 ! added for His-bound Zn

BOND OZ ZN_1 1000.000 {sd= 0.001} 2.000 ! added for Glu-bound Zn

BOND C OZ 1000.000 {sd= 0.001} 1.249 ! added for Glu-bound Zn

ANGLe CH1E C OZ 500.000 {sd= 0.031} 118.0611 ! added for ZN-bound GLU

ANGLe CH2E C OZ 500.000 {sd= 0.031} 118.4969 ! added for ZN bound GLU

ANGLe NR ZN_1 NR 500.000 {sd= 0.031} 109.5000 ! added HIS-ZN-HIS

ANGLe OZ ZN_1 NR 500.000 {sd= 0.031} 109.5000 ! added HIS-ZN-GLU

ANGLe ZN_1 NR CR1E 500.000 {sd= 0.031} 120.0000 ! added HISD-ZN, HISE-ZN

ANGLe ZN_1 NR CRH 500.000 {sd= 0.031} 120.0000 ! added HISD-ZN

ANGLe ZN_1 NR C5 500.000 {sd= 0.031} 120.0000 ! added HISE-ZN

ANGLe ZN_1 OZ C 500.000 {sd= 0.031} 120.0000 ! added ZN-GLU

ANGLe O C OZ 500.000 {sd= 0.031} 123.3548 ! added for ZN bound GLU

IMPRoper C CH2E O OZ 500.000 {sd= 0.031} 0 -0.0137 ! added for ZN bound GLU

IMPRoper NR ZN_1 CR1E CRH 500.000 {sd= 0.031} 0 0.0000 ! added for His-bound Zn(II)

IMPRoper NR C5 CRH ZN_1 500.000 {sd= 0.031} 0 0.0000 ! added for His-bound Zn(II)

DIHEdral OZ C CH2E CH2E MULT 2 1.00 2 0.0000 0.5 6 0.0000 ! added for ZN bound GLU

**Zn^2+^-site topology:**


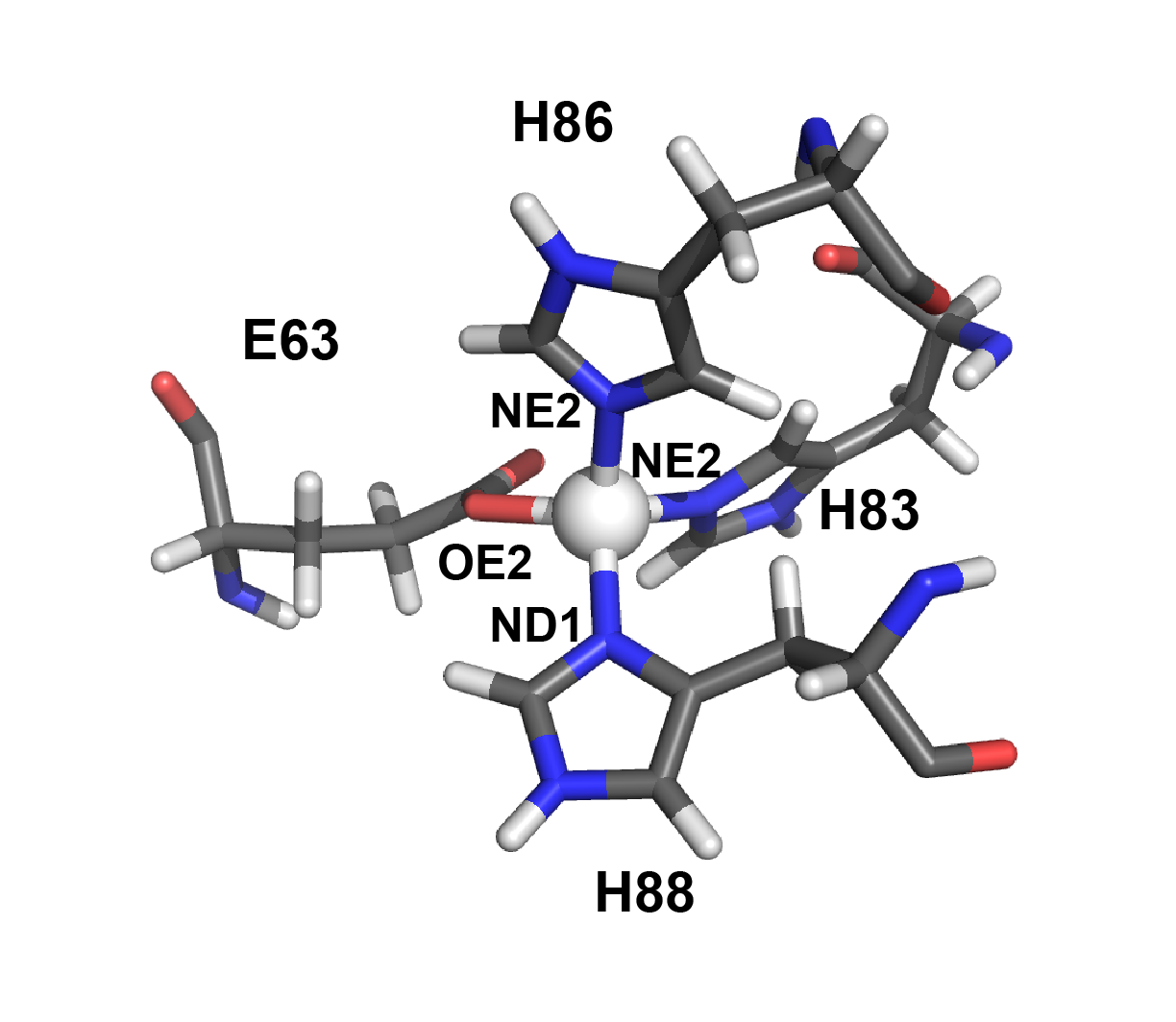

Supplement: Protocol S1 — This protocol describes the construction and the force-field parameters of the non-standard residue for the Zn2+-site using CNS. (DOC) [file pone.0081168.s006.doc]
